# Strongyloides stercoralis infection after the use of emergency corticosteroids: a case report on hyperinfection syndrome

**DOI:** 10.1186/s13256-019-2022-y

**Published:** 2019-04-29

**Authors:** George Vasquez-Rios, Roberto Pineda-Reyes, Eloy F. Ruiz, Angelica Terashima, Fernando Mejia

**Affiliations:** 1 Department of Internal Medicine, Saint Louis University School of Medicine, Saint Louis, MO USA; 2 Laboratory of Parasitology, Tropical Medicine Institute Alexander von Humboldt, Universidad Peruana Cayetano Heredia, Lima, Peru; 3 CONEVID, Unidad de Conocimiento y Evidencia, Universidad Peruana Cayetano Heredia, Lima, Peru

**Keywords:** Asthma, Corticosteroids, Strongyloidiasis, *Strongyloides*, Hyperinfection syndrome, Case report

## Abstract

**Background:**

In clinical practice, identification of a case of severe asthma exacerbation prompts initiation of corticosteroids. However, not all that wheezes is asthma.

**Case presentation:**

A 61-year-old man from the Peruvian Amazon presented with progressive dyspnea, abdominal pain, and cough for the past week. His medical history was remarkable for asthma since childhood; he was treated with beta-agonists, ipratropium, and orally administered corticosteroids. On evaluation, he was febrile and ill-appearing. His chest examination revealed diffuse wheezing and bilateral crackles. He was diagnosed as having community-acquired pneumonia and asthma exacerbation and was started on empiric antibiotics, nebulized beta-agonists, and orally administered corticosteroids. His clinical status continued deteriorating and he became critically ill despite broad-spectrum antibiotics and antifungals. Considering the epidemiological background of our patient, bronchoalveolar and fecal samples were obtained to investigate soil-transmitted helminths. Larvae of *Strongyloides stercoralis* were found in both specimens. Ivermectin was initiated and corticosteroids were discontinued. He experienced remarkable improvement of clinical condition over the next weeks. The literature on this topic was reviewed.

**Conclusion:**

Cases of severe asthma exacerbation warrant careful evaluation before the initiation of corticosteroids, especially in patients at risk for parasitic infections. A high index of suspicion is critical. Alternative etiologies of respiratory decompensation should be considered in patients who fail to improve with broad-spectrum antibiotics and antifungals.

## Background

*Strongyloides stercoralis* is an intestinal nematode with a worldwide distribution [1]. Rural regions in tropical and subtropical countries are known to have a high prevalence of this organism [1, 2]. Travelers, military personnel, and immigrants coming to developed countries can host this parasite for years without expressing any complaint [1–3]. Symptomatic strongyloidiasis may manifest with gastrointestinal (GI) complaints and asthma-like symptoms [4, 5]. However, patients undergoing immunosuppressive therapy or with severely debilitated immune status can develop an unusual phenomenon known as hyperinfection syndrome (HS) [6, 7]. HS is characterized by critical illness and multi-organ dysfunction due to massive dissemination of the parasite [6–8].

Discussions about the association between asthma and *Strongyloides* date back to the 1960s [9–11] and a few cases of patients with asthma suffering exacerbations attributed to *S. stercoralis* infection have been reported in the literature [12–34]. Although the underlying pathophysiology is still unclear, corticosteroid use during asthma exacerbations could trigger severe forms of disease, including *Strongyloides* HS [2–5]. Therefore, HS remains an obscure cause of respiratory distress among individuals with asthma with high mortality rates if the diagnosis is delayed [6–8].

This is a case report of a patient who survived an unusual presentation of hyperinfection by *S. stercoralis*, masquerading as an asthma exacerbation. There are limited data on patients who survived aggressive forms of *Strongyloides* HS especially after receiving high doses of corticosteroids [7]. Therefore, there is a need for further research to optimize current management recommendations. This study aims to provide critical care physicians and internists with clinical clues that may help them arrive at the diagnosis in a timely fashion as well as guide management.

## Review of the literature

**Fig. 1:**
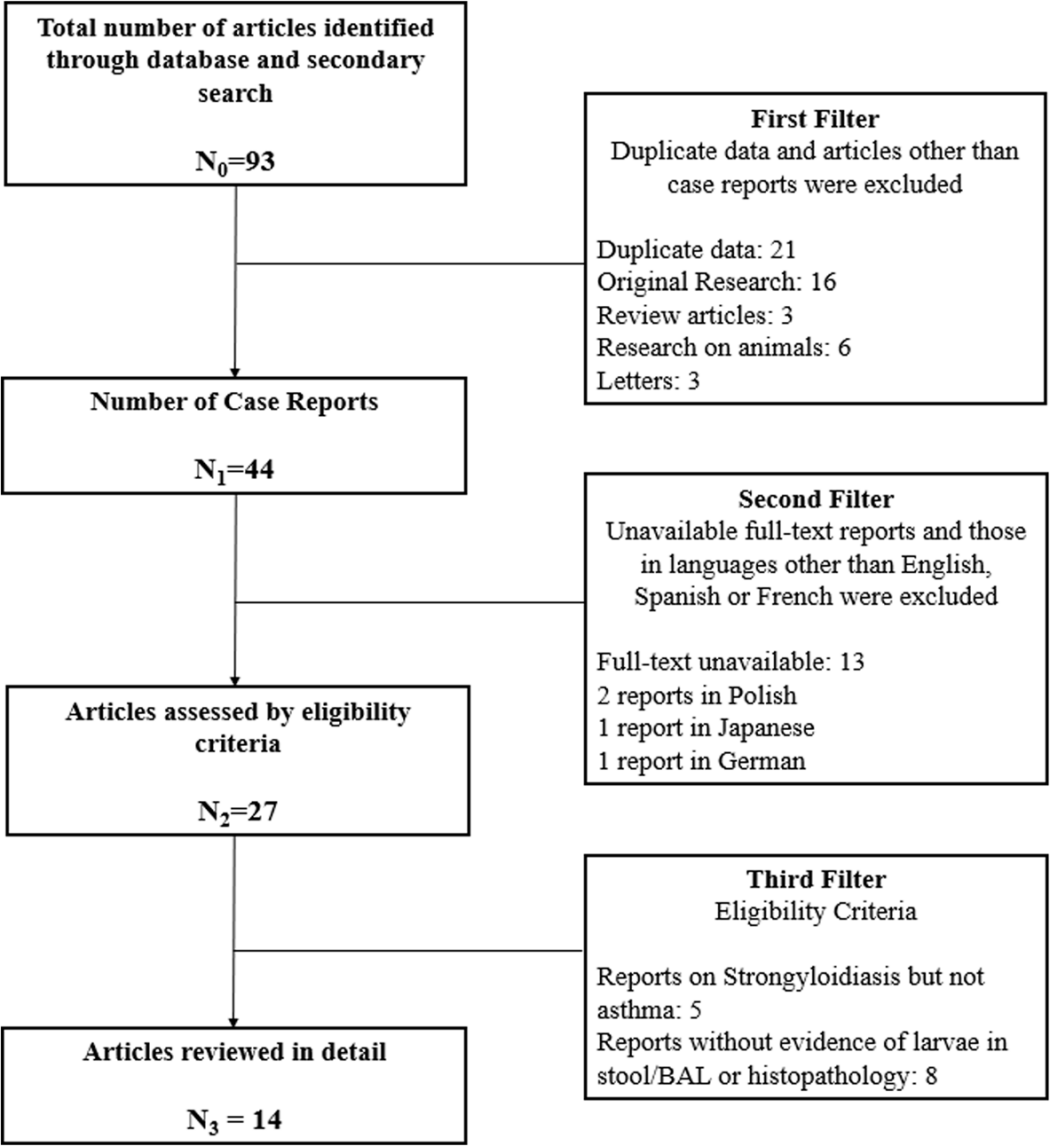
Review of the literature. *BAL* bronchoalveolar lavage

Two independent investigators (GVR and RPR) searched the literature to identify cases of *Strongyloides* HS in patients with asthma. Eligibility criteria were: (1) case reports including individuals with asthma exacerbation who tested positive for *S. stercoralis* by means of any fluid or solid specimen (stool, bronchial secretions); and (2) diagnosis of *Strongyloides* HS based on microscopic visualization of the larvae from bronchial secretions or through histopathology. Abstracts were reviewed in PubMed, EBSCO, LILACS, and SciELO. MeSH search terms included: “*Strongyloides*” AND “asthma”; “*Strongyloides stercoralis*” AND “asthma”; “Strongyloidiasis” AND “asthma”. Also, we conducted a secondary search of the literature through Google/Google Scholar and reviewed previous references. Reports in English, French, or Spanish languages were included. Figure 1 presents the abstract screening process. Reports that met the eligibility criteria were analyzed in detail. The authors evaluated those articles and any discrepancy was solved by consensus. Data were collected in case forms and stored in a spreadsheet.

## Case presentation

A 61-year-old man from the Peruvian Amazon presented to the Emergency Department with a 1-week history of progressive shortness of breath, fever, and cough. His medical background was significant for essential hypertension and asthma. His home medications included lisinopril, fluticasone/salmeterol, ipratropium, and low-dose prednisone. He worked as a farmer in the Peruvian rainforest and had no known environmental exposure to pollutants or toxins. He did not smoke tobacco but he was a former alcohol user who quit drinking alcohol 5 years before presentation. He had a family history of hypertension. On evaluation, he was ill-appearing and in respiratory distress. His vital signs were: temperature (T) 38.2 °C, blood pressure 110/70 mmHg, heart rate 105 beats per minute (bpm), and respiratory rate 28 respirations/minute with saturation of oxygen (SO_2_) 87% on room air. Chest auscultation revealed diffuse wheezing and bilateral crackles. His cardiovascular examination showed tachycardia without gallops or murmurs. Furthermore, his neurological examination was negative for focal deficits or meningeal signs. The rest of the physical examination was unremarkable.

**Fig. 2:**
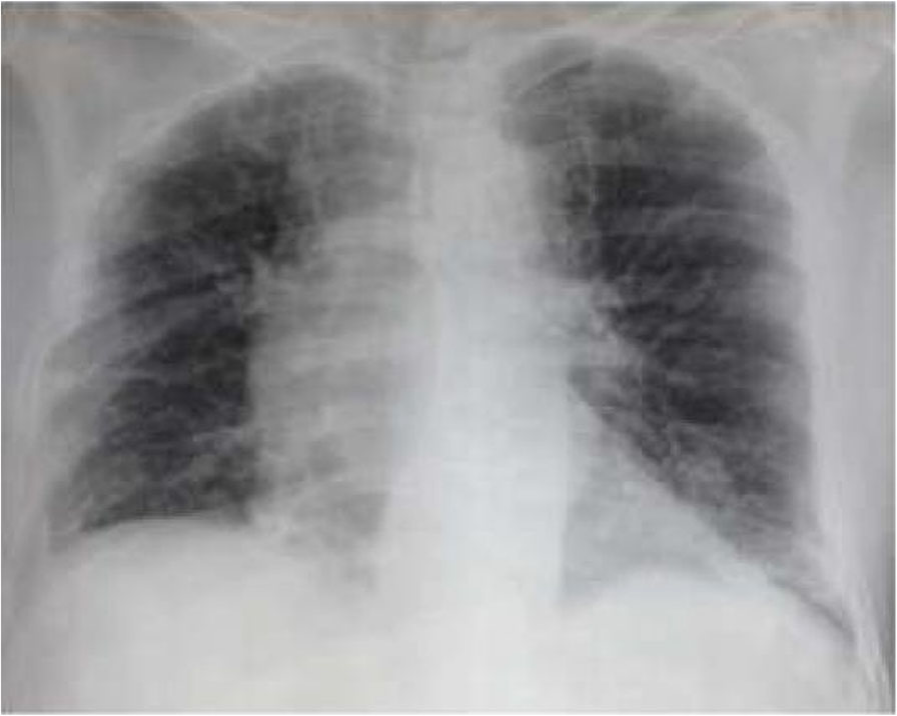
Chest X-ray showing bilateral interstitial infiltrates, predominantly in the bases

**Fig. 3:**
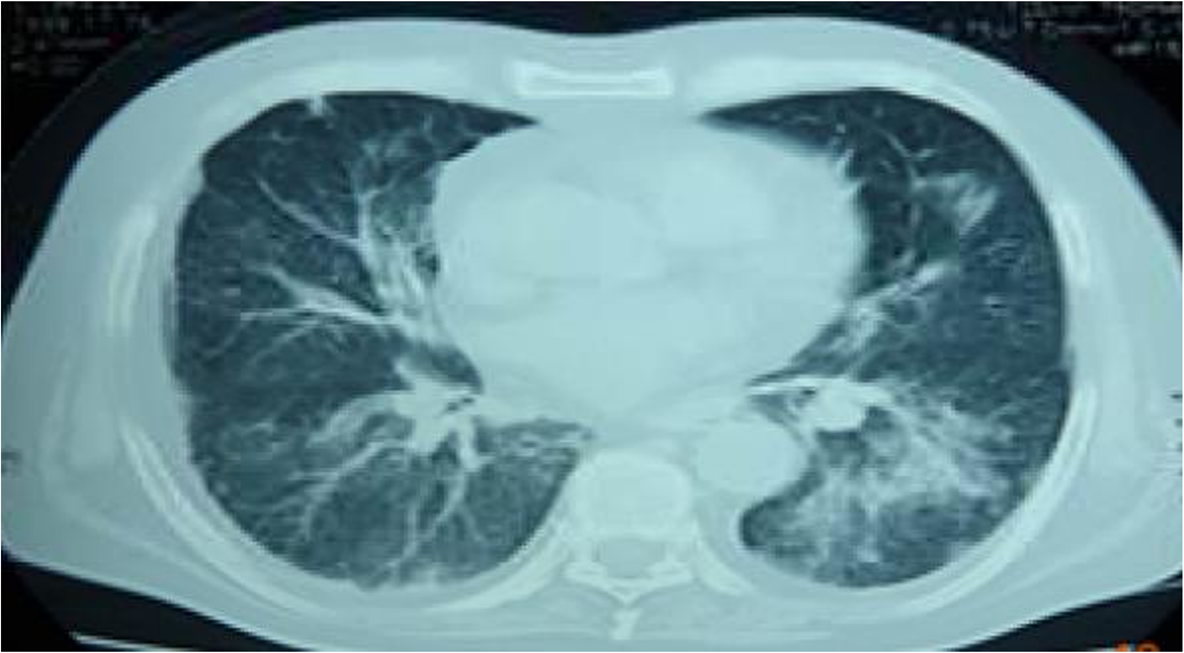
Chest computed tomography scan revealing ground-glass opacities and interstitial infiltrates bilaterally, predominantly in the left side

Initial laboratory results showed a white blood cell count of 34 × 10^9^/L (bands 5%, lymphocytes 1.7%, eosinophils 0.3%). Biochemical analysis revealed hyponatremia, mild elevation of hepatic enzymes, and severe hypoalbuminemia. His arterial blood gases revealed: pH 7.28, partial pressure of carbon dioxide (pCO_2_) 55 mmHg, and partial pressure of oxygen (pO_2_) 59 mmHg. A chest X-ray showed bilateral base-predominant interstitial infiltrates concerning for community-acquired pneumonia (Fig. 2). He was administered ceftriaxone and azithromycin, albuterol nebulization, and biphasic positive airway pressure support. In addition, a dose of prednisone (1 mg/kg) was administered orally for severe obstructive airway disease. He exhibited partial clinical improvement over the following 48 hours, but due to worsening oxygen requirements and persistent fever, his antibiotic therapy was switched to meropenem and vancomycin. Figure 3 shows a computed tomography (CT) scan with bilateral consolidations, predominantly on the lower lobe of his left lung.

**Fig. 4:**
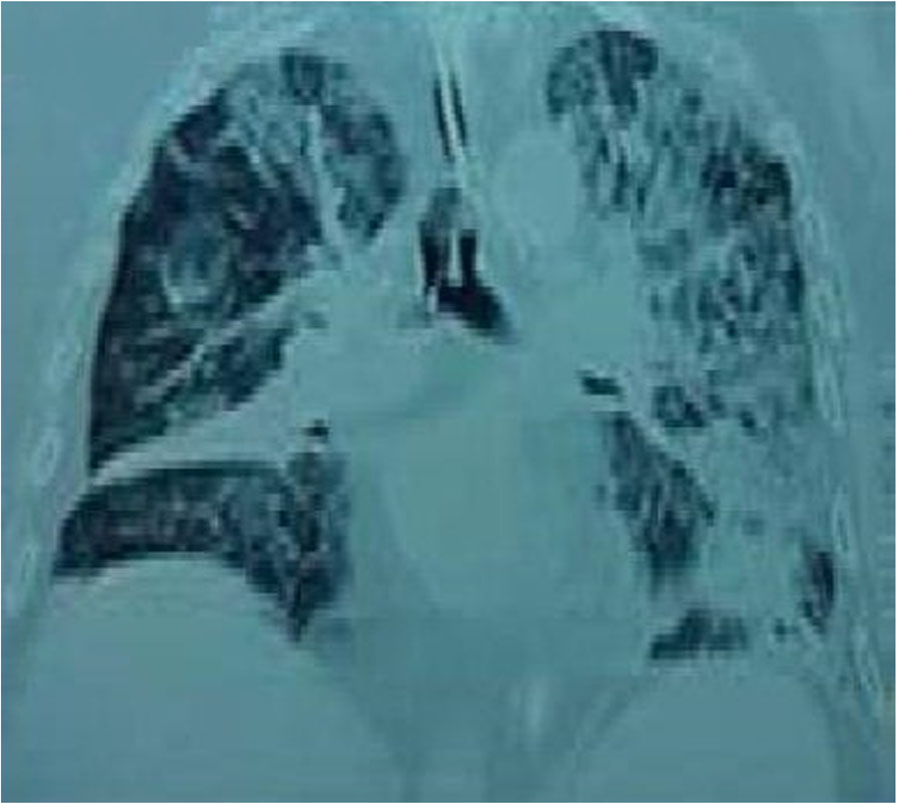
Repeat chest computed tomography scan showing diffuse interstitial infiltrates and consolidations

**Fig. 5:**
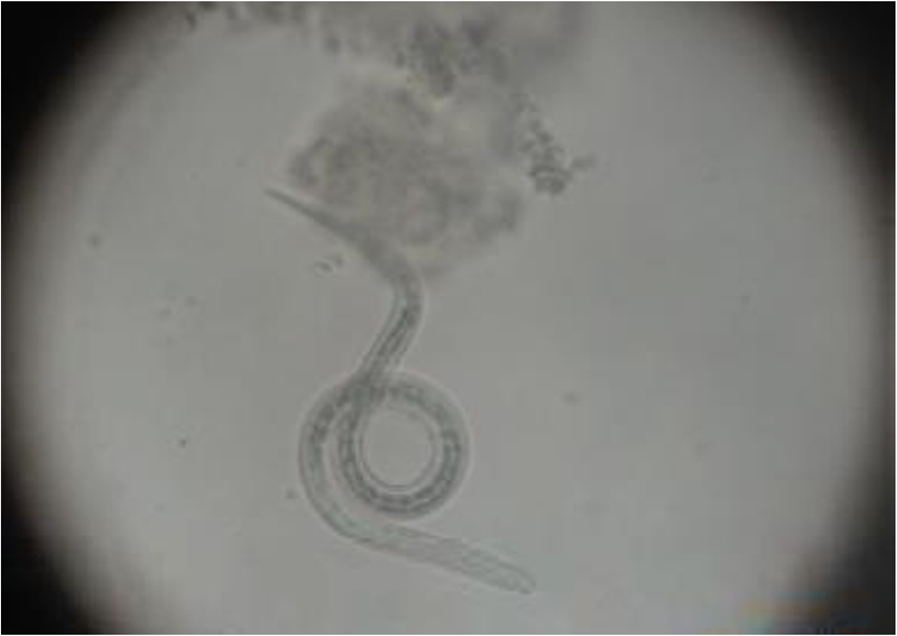
*Strongyloides stercoralis* larvae found in bronchoalveolar lavage

**Fig. 6:**
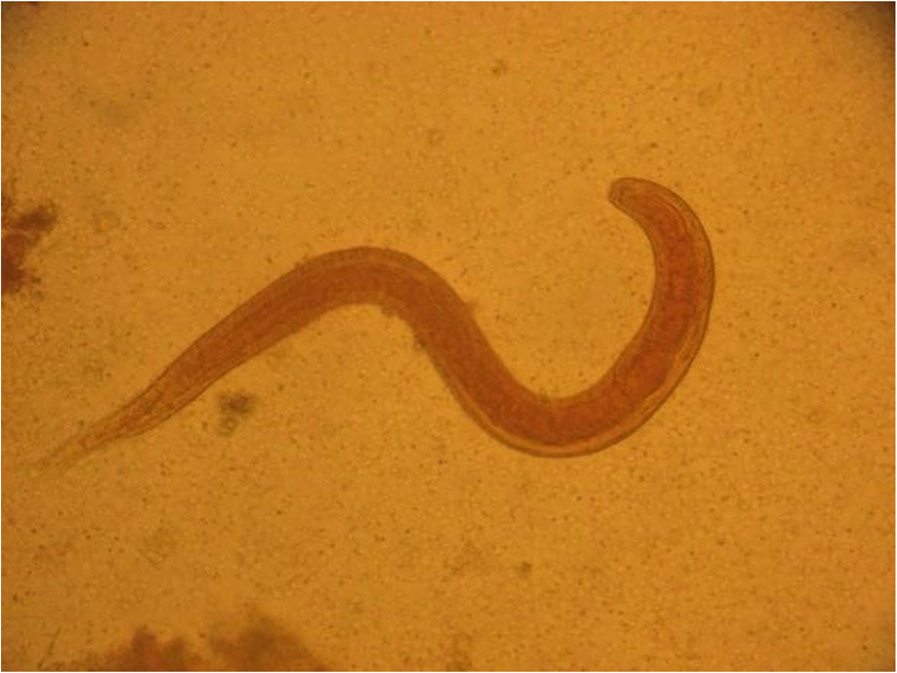
*Strongyloides stercoralis* larvae found in stool examination

On hospital day 5, he presented hemodynamic instability and acute encephalopathy, which prompted intubation and vasopressor support. Arterial blood gases showed: pH 6.9, pCO_2_ 70.8 mmHg, and pO_2_ 51 mmHg. His lactic acid level was 10.7 mmol/L. A repeat chest CT scan revealed extensive bilateral infiltrates and ground-glass opacities (Fig. 4). Empiric therapy with micafungin was initiated. Bronchoalveolar lavage (BAL) was negative for conventional bacteria, fungi, or acid-fast bacilli. An extensive work-up was unremarkable including investigations for HIV and Human T-cell lymphotropic virus (HTLV-1), which were non-reactive. Considering the patient epidemiological background and his rapid deterioration despite broad-spectrum antibiotics, a new BAL was conducted to test for soil-transmitted helminths (STH). Fecal samples were collected as well. Finally, larvae of *S. stercoralis* were found in both BAL (Fig. 5) and stool specimens (Fig. 6). Corticosteroid therapy was discontinued and anti-parasitic treatment was started with ivermectin 200 μg/kg per day orally for 2 days. Therapy was repeated 2 weeks later to ensure adequate parasite eradication. Stool and bronchial specimens were negative thereafter. The patient experienced progressive recovery over the next 4 weeks. Unfortunately, he was lost to follow-up afterward.

## Literature analysis

Fourteen cases of asthma exacerbation associated with *Strongyloides* HS were identified (Table 1). The mean age was 58.6 years. The male-to-female ratio was 5:2. Of the individuals, 21% presented one or more comorbid conditions including ischemic heart disease, Cushing syndrome, and rheumatoid arthritis. All except one were born in endemic areas for *Strongyloides* and one was born in a developed country but served as military personnel in a high-risk country for STH. More than half of the cases (57%) were reports from health care centers in developed countries.

**Table 1:** Hyperinfection syndrome due to *Strongyloides stercoralis* in patients with asthma

Author and year		Age/Sex	Risk factors	Comorbidities	AEC (cells/μL)	Larvae in sputum/BAL	Larvae in stool	Histopathological findings	Time to anti-parasitic administration (days)	Anti-parasitic treatment, duration	Outcome
1	Nwokolo and Imohiosen 1973 [12]	15/F	EEA	None	3502	YES	NO	NA	NA	TBZ, 2 days DEC, 25 days	Unknown
2	Higenbottam and Heard 1976 [13]	57/M	EEA, steroids	None	11	NO	NO	Larvae in bronchial sections of both lungs	None	None	Died
3	Kaslow *et al*. 1990 [14]	74/M	EEA, steroids	None	2665	YES	YES	Larvae in the heart, stomach, and colon	3	TBZ, 17 days	Died
4	Marsan *et al*. 1993 [15]	54/M	EEA, steroids	None	< 130	YES	NA	NA	NA	TBZ	Survived
5	Rivals *et al*. 2000 [16]	60/M	EEA, steroids	Ischemic heart disease	NA	YES	YES	NA	2	TBZ, 6 days	Survived
6	Jones *et al*. 2002 [17]	75/M	EEA, steroids	None	1200	YES	NO	NA	NA	ABZ, 3 days IVM, 11 days	Survived
7	Upadhyay *et al*. 2001 [18]	82/M	EEA, steroids	None	324	YES	NA	Larvae infiltration in lung	5	ABZ, 7 days	Died
8	Ochoa *et al*. 2003 [19]	55/M	EEA, steroids	None	NA	YES	YES	Larvae in mucosa and submucosa of the main bronchi	9	ABZ, 3 days	Died
9	Kim *et al*. 2005 [20]	71/M	EEA, steroids, DM, alcohol use	COPD, Cushing syndrome, DM	165	YES	NO	NA	None	None	Died
10	Jayaprakash *et al*. 2009 [21]	50/F	EEA, steroids	None	NA	YES	YES	NA	NA	IVM, 7 days	Survived
11	Altintop *et al*. 2010 [22]	68/F	EEA, steroids, methotrexate	RA	NA	YES	YES	Larvae in gastric glands and duodenal crypts	NA	ABZ, 23 days IVM, 3 days	Survived
12	Bashir *et al*. 2015 [23]	70/M	EEA, steroids, DM	DM	109	YES	YES	NA	NA	IVM, 2 days	Survived
13	Alsharif *et al*. 2015 [24]	31/F	EEA, steroids	None	3831	YES	YES	NA	NA	IVM, 17 days	Survived
14	Wang *et al*. 2016 [25]	NA/M	EEA, steroids	None	1099	YES	YES	Larvae in duodenal mucosa	NA	ABZ, 14 days	Died

*ABZ* albendazole, *AEC* absolute eosinophil count, *BAL* bronchoalveolar lavage, *COPD* chronic obstructive pulmonary disease, *DEC* diethylcarbamazine, *DM* diabetes mellitus, *EEA* exposure to an endemic area, *F* female, *IVM* ivermectin, *M* male, *NA* not available, *RA* rheumatoid arthritis, *TBZ* thiabendazole

Among clinical manifestations, 64% of individuals presented with respiratory distress and 21% with GI symptoms. Fever was inconsistently reported. Two presented hemoptysis. Eosinophilia was reported in 20% of the cases that included this laboratory result (10 reports), while hypereosinophilia was seen in 30% of them. BAL and/or sputum were positive in 93% of cases, and the remaining individuals were diagnosed with *Strongyloides* HS by lung histopathology, which yielded numerous larvae during the autopsy. Stool examination was positive for larvae of *Strongyloides* in 67% of those patients tested (8 out of 12). Anti-parasitic therapy was administered to 86% of individuals (12 out of 14); ivermectin was the drug of choice in 42% of the cases (5 out of 12). Two of those patients received ivermectin plus another anti-parasitic drug. Overall, the mortality rate was 46% (6 out of 13; 1 outcome was unknown). Notably, individuals treated with ivermectin alone or in combination therapy had a survival rate of 100% (five out of five), while only 33% (two out of six) of those treated with a different anti-parasitic drug survived.

## Discussion

This case report presents a patient with symptoms related to bronchial hyperresponsiveness, presumed to be an asthma exacerbation complicated by pneumonia and sepsis. Mild or absent eosinophilia with severe obstructive respiratory symptoms may lead clinical judgment toward common etiologies. However, a high degree of suspicion along with consideration of this patient’s epidemiologic background were vital for appropriate investigation of the presence of *S. stercoralis* in BAL and stool samples. Compared to previous literature, this is an unusual case of a patient who survived septic shock, *Strongyloides* HS, and high-dose steroid therapy.

Strongyloidiasis has become an emergent disease in developed countries because of immigration. Ostera *et al.* detected a prevalence of 4.2% among Latin American immigrants in Washington DC, USA [34]. More than half of the cases included in the literature review (Table 1) were reported in developed countries including the USA, England, France, and South Korea [13–18, 20, 24]. However, all the patients were exposed to endemic areas. This is an important finding, since the popularization of traveling and globalization could favor the presentation of *Strongyloides* in non-endemic areas, a trend noticed over the last 20 years [2, 5].

Clinical manifestations of *S. stercoralis* infection may range from asymptomatic to chronic symptoms, and *Strongyloides* HS with multiple systems involved [2, 4]. *Strongyloides* HS is the result of the high replication and migration of the larvae, typically seen in patients with impaired cell-mediated immunity as in those with HTLV-1 infection, transplant recipients, or individuals on chronic or high-dose corticosteroids [2, 4, 7, 8]. On the other hand, HIV has not been frequently associated with *Strongyloides* HS [2, 3]. It usually presents with fever and GI complaints (for example, nausea, vomiting, abdominal pain, diarrhea, bleeding); however, extra-intestinal manifestations are also common, including dyspnea, wheezing, pulmonary infiltrates, or alveolar hemorrhage [2–4]. *Strongyloides* HS may be complicated with shock, disseminated intravascular coagulation, and respiratory failure [2, 4, 7, 8]. Interestingly, the main presentation of *Strongyloides* HS noticed in the literature review, as well as in this case, was pulmonary involvement (64%) as opposed to the expected GI manifestation caused by helminths.

*Strongyloides* infection in healthy individuals characteristically produces marked eosinophilia [2–5]. In contrast, patients with *Strongyloides* HS may present with a higher number of larvae but few eosinophils [2, 5, 8]. Increased eosinophil count was observed in half of cases with available data (five out of ten patients); and it was present only in one third of patients who died. Our patient did not have eosinophilia. As stated before, corticosteroid therapy is one of the most frequent risk factors for *Strongyloides* HS in developed countries and may contribute to an adverse outcome [5, 7]. Corticosteroids affect T-helper type-2 (Th2) response and eosinophil migration to the site of infection. Also, there is evidence suggesting that corticosteroids can play a role as molting signals for eggs, which enhances parasite production and promotes dissemination [2–5].

Although malnutrition and HTLV-1 infection are strong risk factors for strongyloidiasis in developing countries [2], this information was unavailable from the literature review. The patient had low albumin levels, probably reflecting his defective nutrition. He also tested negative for HIV and HTLV-1. The latter is especially important in *Strongyloides* HS, as it may induce a predominant Th1 cell response with high levels of interferon-γ (IFN-γ) and tumor necrosis factor-α (TNF-α). Furthermore, there is a decreased Th2 response that impairs the secretion of interleukin (IL)-4, IL-13, IL-5, and Ig E, which further reduces eosinophil recruitment and blunts the normal inflammatory response against the parasite [3, 5]. Thus, investigation of HTLV-1 status is mandatory in patients with strongyloidiasis.

Examinations revealing larvae in bronchial fluids are an important criterion for confirming *Strongyloides* HS and dissemination [5]. Traditional stool-based techniques have low sensitivity (< 50%) for *Strongyloides* detection [35] and require multiple repeated examinations or concentration methods (such as Baermann) to increase its sensitivity [2, 3, 7]. However, it is hypothesized that in cases of *Strongyloides* HS, the larvae output is higher and the sensitivity of stool-based methods may increase [2, 3]. In this review, one third of the cases had negative stool samples (4 out of 12), which could be explained by deficient sample collection in critically ill patients, insufficient number of stool samples provided for analysis, or lack of laboratory expertise. Molecular-based studies including enzyme-linked immunosorbent assay (ELISA) tests have the highest sensitivity (near 100%) [36]; however, their availability, cost, and time-to-result may limit their role in an intensive care unit.

Suspicion of *Strongyloides* HS warrants immediate anti-parasitic treatment since mortality rates may be as high as 87% [6]. A recommended regimen is ivermectin 200 μg/kg per day for 2 days, repeated during the second and fourth week [3]. A single dose of ivermectin 200 μg/kg has been demonstrated to be superior to a 7-day course of albendazole 800 mg (93% versus 63% success rate) [37]. In this review, three patients who received albendazole but not ivermectin ultimately expired. Two patients who did not receive any anti-parasitic drug also died. However, 100% of the individuals who received ivermectin alone or in combination with another anti-parasitic drug survived. Overall mortality rate was 46% in our patient cohort, which is concordant with the previously reported literature (15–87%) [5]. There is limited evidence for the use of parenteral ivermectin and its use is restricted in clinical practice.

## Conclusion

*Strongyloides* HS should be suspected in critically ill patients with asthma with a pertinent epidemiological background, and for whom conventional breathing therapy and broad-spectrum antibiotics failed. Once the diagnosis of *Strongyloides* infection is established, corticosteroids should be discontinued and therapy with ivermectin should be initiated promptly. A high index of suspicion and epidemiological risk assessment are the cornerstone for the diagnosis of this condition in both developed and developing countries.

## Abbreviations

BAL: Bronchoalveolar lavage
bpm: Beats per minute
CT: Computed tomography
ELISA: Enzyme-linked immunosorbent assay
GI: Gastrointestinal
HIV: Human immunodeficiency virus
HS: Hyperinfection syndrome
HTLV-1: Human T-cell lymphotropic virus
IFN-γ: interferon-γ
IL: Interleukin
pCO_2_: Partial pressure of carbon dioxide
pO_2_: Partial pressure of oxygen
SO_2_: Saturation of oxygen
STH: Soil-transmitted helminths
T: Temperature
Th2: T-helper type-2
TNF-α: Tumor necrosis factor-α
